# Activation of the prostaglandin E_2_ EP_2_ receptor attenuates renal fibrosis in unilateral ureteral obstructed mice and human kidney slices

**DOI:** 10.1111/apha.13291

**Published:** 2019-05-20

**Authors:** Michael Schou Jensen, Henricus A. M. Mutsaers, Stine Julie Tingskov, Michael Christensen, Mia Gebauer Madsen, Peter Olinga, Tae‐Hwan Kwon, Rikke Nørregaard

**Affiliations:** ^1^ Department of Clinical Medicine Aarhus University Aarhus Denmark; ^2^ Department of Urology Aarhus University Hospital Aarhus Denmark; ^3^ Department of Pharmaceutical Technology and Biopharmacy University of Groningen Groningen the Netherlands; ^4^ Department of Biochemistry and Cell Biology, School of Medicine Kyungpook National University Daegu Korea

**Keywords:** butaprost, cyclooxygenase‐2, precision‐cut kidney slices, prostaglandin E_2_ receptor, renal fibrosis

## Abstract

**Aim:**

Renal fibrosis plays a pivotal role in the development and progression of chronic kidney disease, which affects 10% of the adult population. Previously, it has been demonstrated that the cyclooxygenase‐2 (COX‐2)/prostaglandin (PG) system influences the progression of renal injury. Here, we evaluated the impact of butaprost, a selective EP_2_ receptor agonist, on renal fibrosis in several models of kidney injury, including human tissue slices.

**Methods:**

We studied the anti‐fibrotic efficacy of butaprost using Madin‐Darby Canine Kidney (MDCK) cells, mice that underwent unilateral ureteral obstruction and human precision‐cut kidney slices. Fibrogenesis was evaluated on a gene and protein level by qPCR and Western blotting.

**Results:**

Butaprost (50 μM) reduced TGF‐β‐induced fibronectin (FN) expression, Smad2 phosphorylation and epithelial‐mesenchymal transition in MDCK cells. In addition, treatment with 4 mg/kg/day butaprost attenuated the development of fibrosis in mice that underwent unilateral ureteral obstruction surgery, as illustrated by a reduction in the gene and protein expression of α‐smooth muscle actin, FN and collagen 1A1. More importantly, a similar anti‐fibrotic effect of butaprost was observed in human precision‐cut kidney slices exposed to TGF‐β. The mechanism of action of butaprost appeared to be a direct effect on TGF‐β/Smad signalling, which was independent of the cAMP/PKA pathway.

**Conclusion:**

In conclusion, this study demonstrates that stimulation of the EP_2_ receptor effectively mitigates renal fibrogenesis in various fibrosis models. These findings warrant further research into the clinical application of butaprost, or other EP_2_ agonists, for the inhibition of renal fibrosis.

## 1 INTRODUCTION

Chronic kidney disease (CKD) affects approximately 10% of the adult population in developed countries.^1^ Moreover, the global incidence of CKD is on the rise, and as a consequence the disease greatly impacts health care budgets. Renal fibrosis, which is characterized by the excessive production and deposition of extracellular matrix proteins by activated myofibroblasts, plays a pivotal role in the development and progression of CKD as well as in renal transplant failure.^2^ Fibrosis results in the loss of organ architecture and function, and is regarded as the most damaging process in CKD; yet, despite overwhelming efforts, effective therapeutic targets have not been identified. Thus, an urgent and unmet clinical need remains.

Previously, it has been demonstrated that the cyclooxygenase‐2 (COX‐2)/prostaglandin (PG) system plays a dominant role in the progression of renal injury.^3^, ^4^, ^5^ COX enzymes catalyze the conversion of arachidonic acid into prostaglandins, including prostaglandin E_2_ (PGE_2_), which is an important mediator of numerous physiological processes in the kidney, including renal hemodynamics as well as water and salt balance.^3^ PGE_2 _exerts its biological activity by activating several G protein‐coupled prostanoid receptors, known as EP_1_‐EP_4._ 6 Several studies have demonstrated an important role for the EP_1_‐EP_4_ receptors in renal injury. Previously, it has been reported that EP_1_ deletion in mice reduced diabetes‐induced expression of the fibrotic markers fibronectin and α‐actin.^7^ Furthermore, EP_1_ antagonism, using ONO8711, decreased fibronectin (FN) expression in mouse proximal tubule cells.^7^ In addition, deletion of EP_2_ increases baseline systolic blood pressure and causes salt‐sensitive hypertension, which is a known risk factor for renal damage.^8^ Interestingly, renal gene expression of both EP_2_ and EP_4 _is shown to be increased during renal fibrogenesis, suggesting that these receptors might play a protective role in the fibrotic process.^4^, ^9^ This notion is supported by the fact that butaprost, a selective EP_2_ agonist, inhibits TGF‐β1‐induced myofibroblast transition of human foetal lung fibroblasts.^10^ However, the efficacy of butaprost for the treatment of renal fibrosis remains to be elucidated.

In the current study, we investigated the impact of butaprost on renal fibrogenesis at the cell, tissue and organ levels using well‐established in vitro and in vivo models as well as a recently developed human model of renal fibrosis, *viz.* precision‐cut kidney slices (PCKS). This model is suitable for studying multicellular (pathological) processes, eg, fibrosis, directly in human tissues since cellular heterogeneity as well as organ architecture are maintained in the slices.

## 2 RESULTS

### 2.1 The EP_2_ agonist, butaprost, mitigates TGF‐β‐induced epithelial‐mesenchymal transition (EMT)

**Figure 1:**
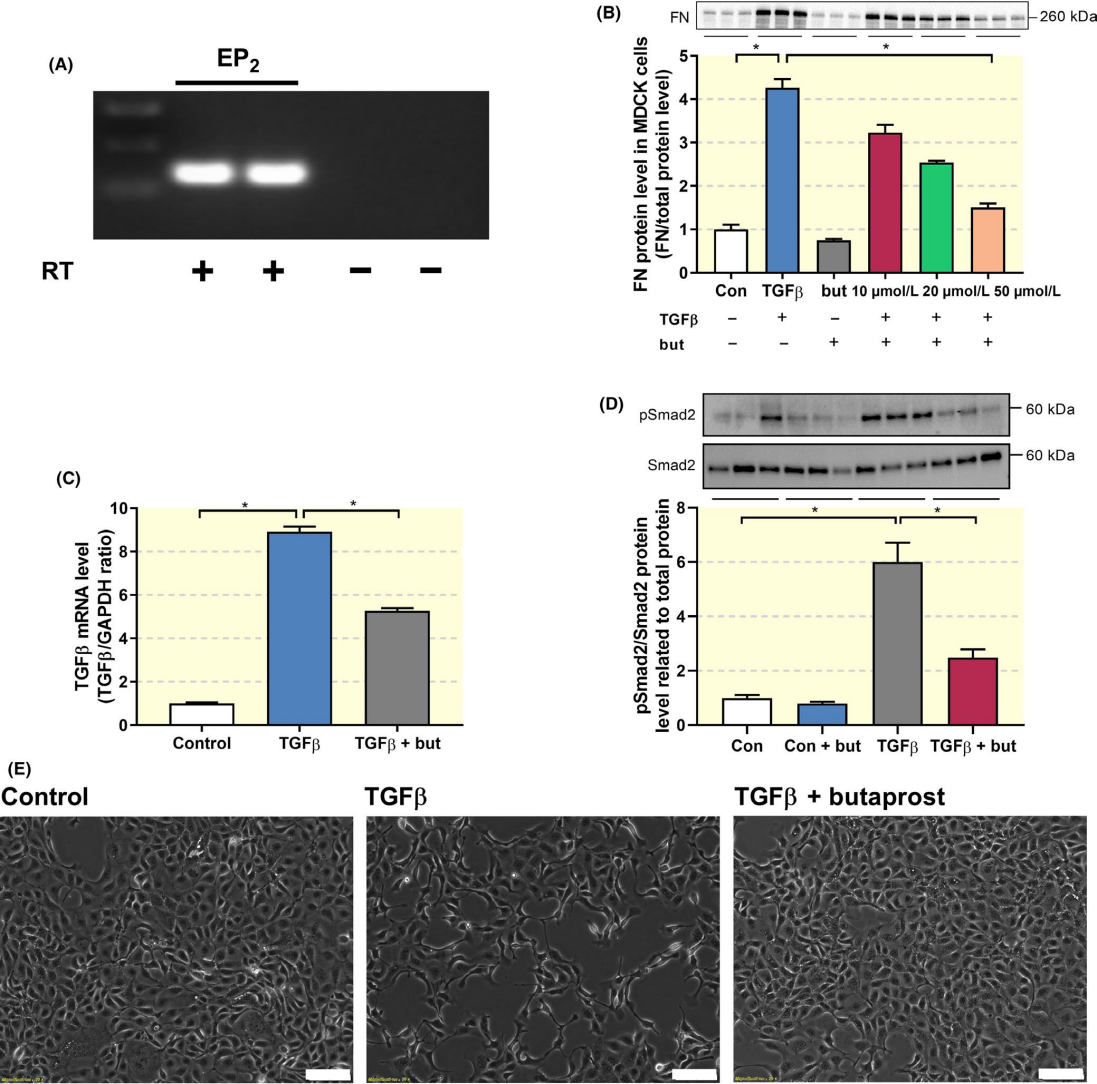
Butaprost attenuates TGF‐β‐induced epithelial‐mesenchymal transition. (A) Gene expression was studied by RT‐PCR with (+) or without (−) reverse transcriptase (RT) enzyme. (B) MDCK cells were exposed to 5 ng/ml TGF‐β in the absence or presence of butaprost (10‐50 μM) for 24 h. FN protein expression was studied using western blotting (n = 3). (C) Gene expression was studied by qPCR. Relative expression was calculated using the reference gene GAPDH (n = 6). (D) Immunoblot analysis of the expression of pSmad2/Smad2 normalized to total protein (n = 6). (E) Representative microscopy images showing MCKD cell morphology. 10× magnification, scale bar is 100 μm. Data are presented as mean ± SEM. *P < 0.05

EMT is an integral part of the fibrotic process. Therefore, we evaluated the impact of butaprost on TGF‐β‐induced EMT in Madin‐Darby Canine Kidney (MDCK) cells, which express the EP_2 _receptor (Figure 1A). As shown in Figure 1B, exposure of MDCK cells to TGF‐β caused a fourfold increase in FN protein expression, which was concentration‐dependently inhibited by butaprost. At the highest tested concentration (50 μM), butaprost almost completely blocked TGF‐β‐induced FN expression. Therefore, this concentration was used for the remainder of the study. In addition, Figure 1 demonstrates that treatment with TGF‐β increased TGF‐β gene expression, stimulated Smad2 phosphorylation and induced a spindle‐like morphology indicative of EMT, all of which could be inhibited by butaprost. Taken together, these findings indicate that butaprost mitigates TGF‐β/Smad signalling and EMT in MDCK cells.

**Figure 2:**
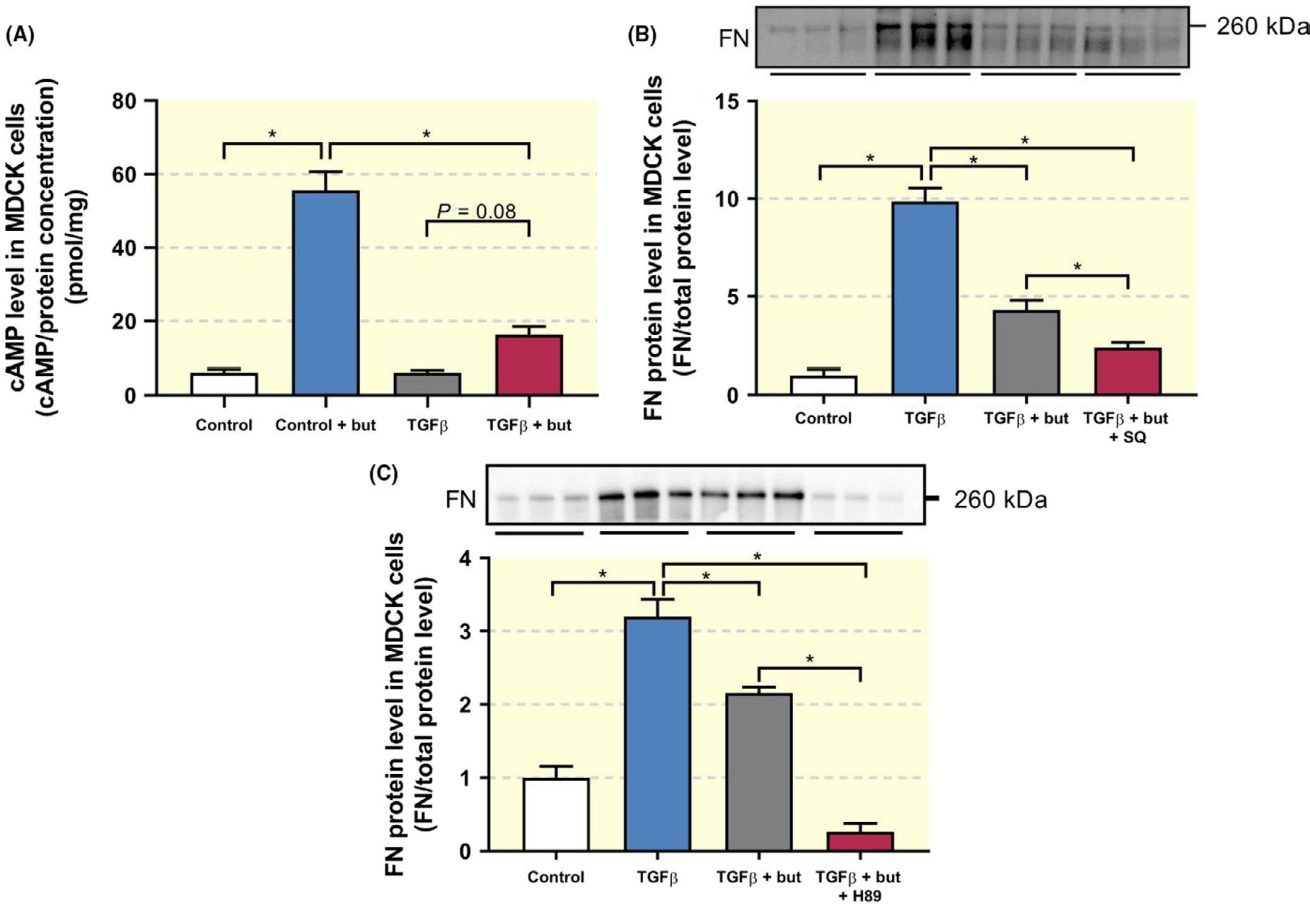
Anti‐fibrotic effect of butaprost is independent of cAMP/PKA signalling. MDCK cells were exposed to 5 ng/ml TGF‐β in the absence or presence of butaprost (50 μM), SQ22536 (75 µM) or H89 (10 µM) for 24 h. (A) cAMP levels were determined in cell lysates via ELISA (n = 6). (B, C) FN protein expression was studied using western blotting (n = 5‐6). Data are presented as mean ± SEM. *P < 0.05

Next, we investigated whether butaprost mitigated the pro‐fibrotic effects of TGF‐β via the cAMP pathway, which has been shown to play a role in pulmonary fibrosis.^10^ It has been demonstrated that activation of the EP_2 _receptor increases intracellular cAMP levels.^3^ Indeed, treatment with butaprost markedly increased intracellular cAMP levels; however, this response was suppressed in presence of TGF‐β (Figure 2A). Since butaprost clearly affected cAMP levels, we evaluated whether this effect was due to changes in adenylate cyclase (AC) activity, the enzyme that converts ATP into cAMP. As shown in Figure 2B, exposure of MDCK cells to a combination of TGF‐β, butaprost and SQ22536 (an AC inhibitor) did not hamper the anti‐fibrotic effect of butaprost.

As inhibition of AC did not attenuate the effects of butaprost, we investigated if protein kinase A (PKA), the cAMP‐dependent activator of cAMP response element‐binding protein (CREB), was involved in its activity. Exposure of MDCK cells to a combination of TGF‐β, butaprost and H89 (a PKA inhibitor) did not reverse the anti‐fibrotic effect of butaprost (Figure 2C). These findings suggest that the impact of butaprost on fibrogenesis is unconstrained by the cAMP/PKA signalling pathway.

### 2.2 Butaprost attenuates unilateral ureteral obstruction (UUO)‐induced fibrosis in mice

**Figure 3:**
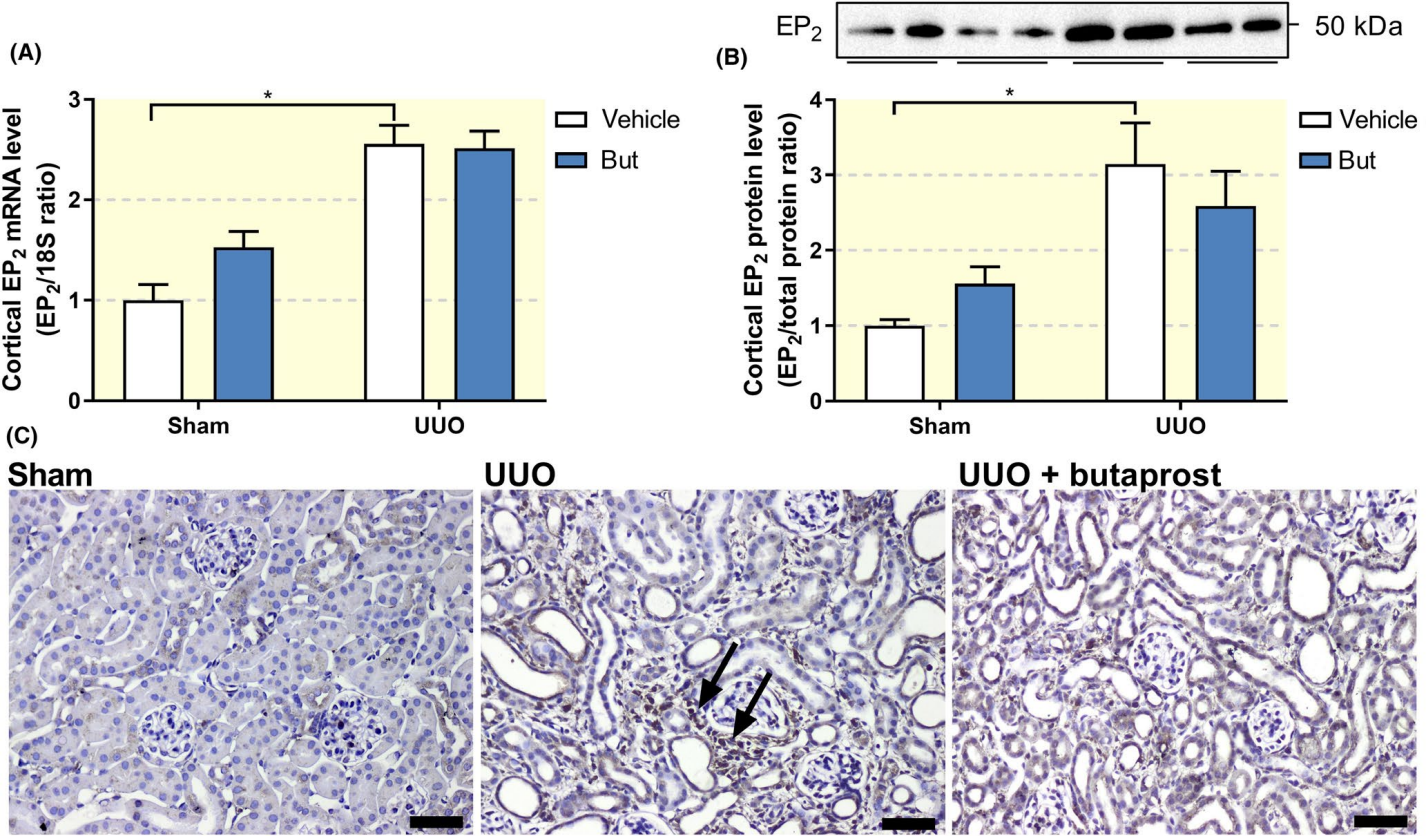
Impact of UUO and butaprost on EP_2_ receptor expression in vivo. Mice were subjected to 7 days of UUO and treated with butaprost (4 mg/kg). (A) Gene expression was studied by qPCR. Relative expression was calculated using the reference gene 18S (n = 6‐10). (B) Cortical EP_2_ protein expression was studied using western blotting (n = 6‐10). (C) Representative immunohistochemistry images showing EP_2_ expression. 20× magnification, scale bar is 50 μm. Arrows indicate EP_2_‐positive interstitial cells. Data are presented as mean ± SEM. *P < 0.05

**Figure 4:**
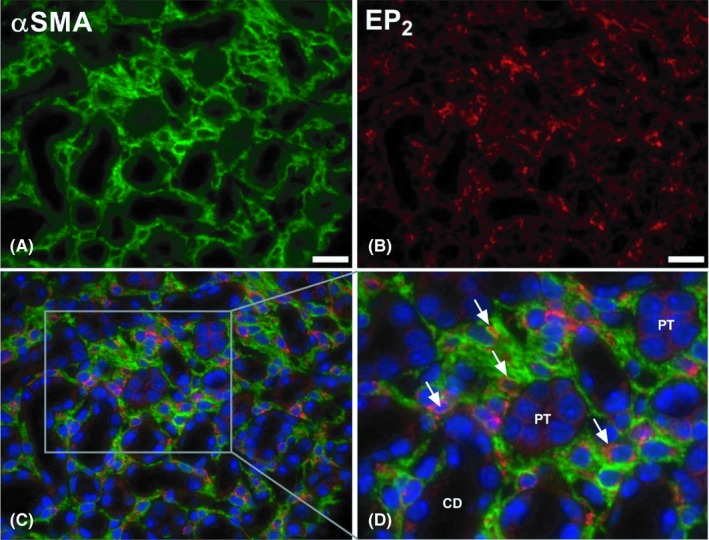
Expression of the EP_2_ receptor in fibrotic renal tissue. Mice were subjected to 7 days of UUO. Afterwards, the UUO kidney was harvested and used for fluorescence microscopy. Representative image of immunolabeling for (A) αSMA (green) and (B) the EP_2 _receptor (red). (C, D) Representative image of co‐immunolabeling (αSMA, green; EP_2 _receptor, red) counterstained with DAPI (blue). 40× magnification, scale bar is 20 μm. Arrows indicate EP_2_‐positive myofibroblasts. CD = collecting duct, PT = proximal tubule

Butaprost clearly reduced fibrogenesis in MDCK cells; therefore, we studied the anti‐fibrotic efficacy of this compound in a murine in vivo model of renal fibrosis, namely UUO. Following surgery, we did not observe any changes in body weight in the four groups. However, the obstructed kidney from UUO mice appeared to be swollen and was increased in weight as compared to sham mice (Table 1). Administration of butaprost did not affect the weight of the obstructed kidney. In addition, plasma creatinine, BUN as well as plasma sodium and potassium did not change between the four groups (Table 1). Next, we confirmed the presence of the EP_2_ receptor in the model using both qPCR and immunohistochemistry. After seven days of UUO, expression of the EP_2_ receptor markedly increased, both on mRNA and protein level (Figure 3). However, this was not significantly altered by butaprost treatment. Immunohistochemical staining of kidney sections revealed that EP_2_ receptor immunoreactive protein was stronger in the UUO kidneys, as compared to sham‐operated mice, and localized to the interstitial cells (Figure 3C). To examine whether the increased EP_2_ receptor labelling was associated with myofibroblasts in the interstitium, we performed double immunofluorescent labelling with antibodies against the EP_2_ receptor (red) and the myofibroblast marker αSMA (green) in the obstructed kidney. As shown in Figure 4, EP_2_ receptor expression co‐localizes with αSMA indicating that the EP_2_ receptor is associated with interstitial myofibroblasts.

**Table 1:** Functional data after UUO and butaprost treatment

Groups	Sham	Sham + butaprost	UUO	UUO + butaprost
Bodyweight (BW) (g)	22.2 ± 0.6	22.8 ± 0.3	22.1 ± 0.5	22.1 ± 0.3
Obstructed kidney/BW (mg/mg mice)	5.8 ± 0.1	6.3 ± 0.3	7.5 ± 0.4*	8.4 ± 0.1#
Creatinine (µmol/L)	11.7 ± 0.8	10.6 ± 0.8	12.8 ± 1	11.3 ± 0.6
BUN (mmol/L)	7.5 ± 0.2	6.3 ± 0.4	8.7 ± 0.6	7.7 ± 0.5
Na (mmol/L)	149.6 ± 0.6	149.5 ± 0.5	150 ± 0.7	149.8 ± 0.4
K (mmol/L)	4.8 ± 0.1	4.8 ± 0.3	4.4 ± 0.1	4.5 ± 0.1

Values are presented as mean ± SEM. Sham: n = 6, sham + butaprost: n = 6, UUO: n = 8 and UUO + butaprost: n = 10.

* *P* < 0.05 compared to sham;

^#^ *P* < 0.05 compared to sham + butaprost.

**Figure 5:**
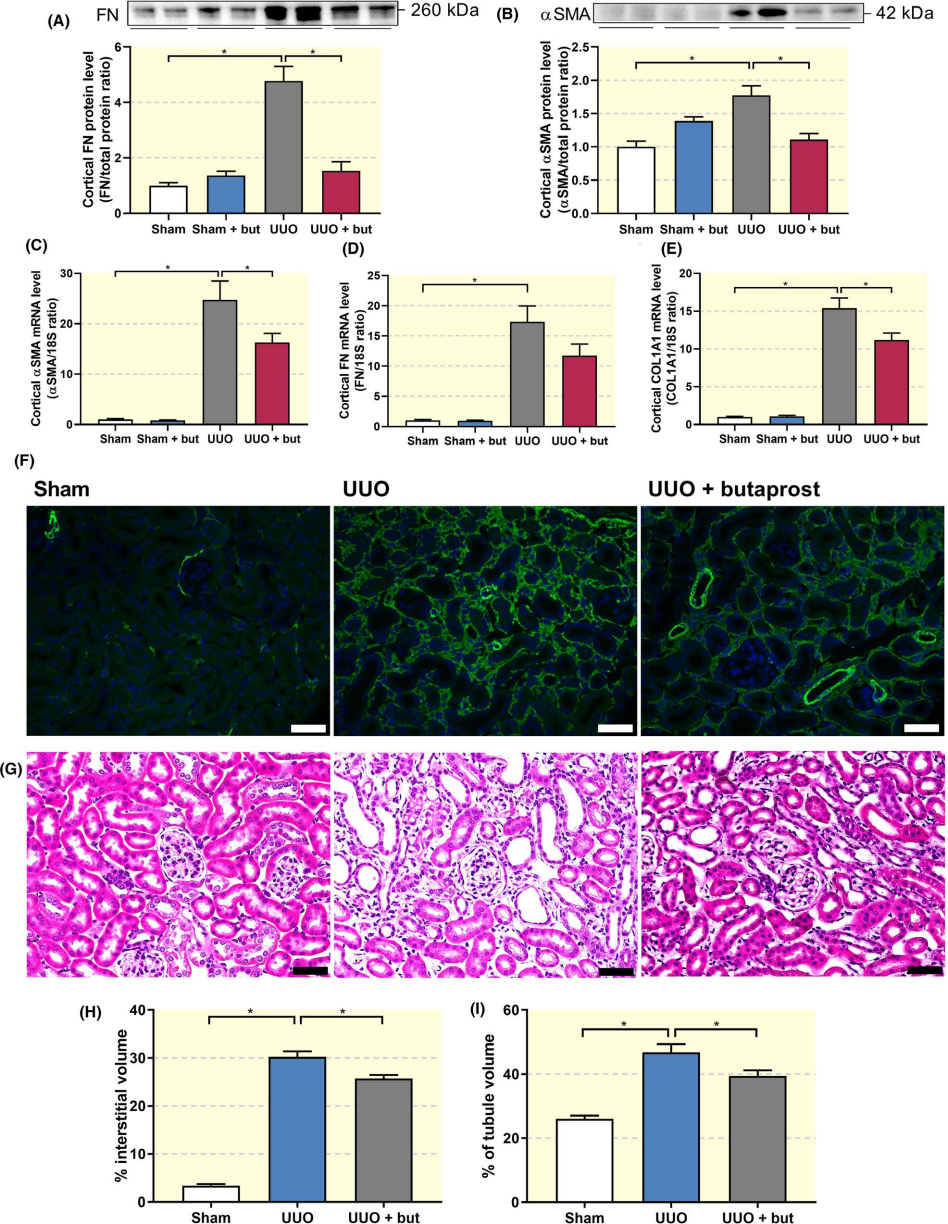
Butaprost mitigates fibrosis in UUO mice. Mice were subjected to 7 days of UUO and treated with butaprost (4 mg/kg). (A) FN and (B) αSMA protein expression was studied using Western blot (n = 6). (C‐E) Gene expression was studied by qPCR. Relative expression was calculated using the reference gene 18S (n = 6‐10). (F) Representative images of immunolabeling for αSMA (green) counterstained with DAPI (blue). 20× magnification, scale bar is 50 μm. (G) Hematoxylin and eosin staining of renal cortical tissue. 20× magnification, scale bar is 50 μm. Quantification of (H) interstitial and (I) tubular volume (n = 4). Data are presented as mean ± SEM. *P < 0.05

Regarding the development of fibrosis, we observed a clear increase in the protein expression of FN and αSMA following UUO, and treatment with butaprost reverted the expression to sham levels (Figure 5A,B). Furthermore, qPCR revealed that 7 days of UUO caused a 25‐fold increase in the gene expression of αSMA, a 17‐fold increase in FN gene expression and a 15‐fold increase in COL1A1 gene expression. Treatment with butaprost significantly reduced the mRNA levels of both αSMA and COL1A1 (Figure 5C‐E). In accordance, fluorescence microscopy revealed that UUO resulted in increased αSMA staining, which could be mitigated by treatment with butaprost (Figure 5F). Furthermore, as shown in Figure 5G‐I, UUO increased both interstitial and tubular volume, indicative of renal damage, which was prevented by butaprost treatment. Thus, stimulation of the EP_2 _receptor attenuates UUO‐induced renal fibrosis in mice.

### 2.3 Stimulation of the EP_2_ receptor mitigates fibrogenesis in human PCKS

**Figure 6:**
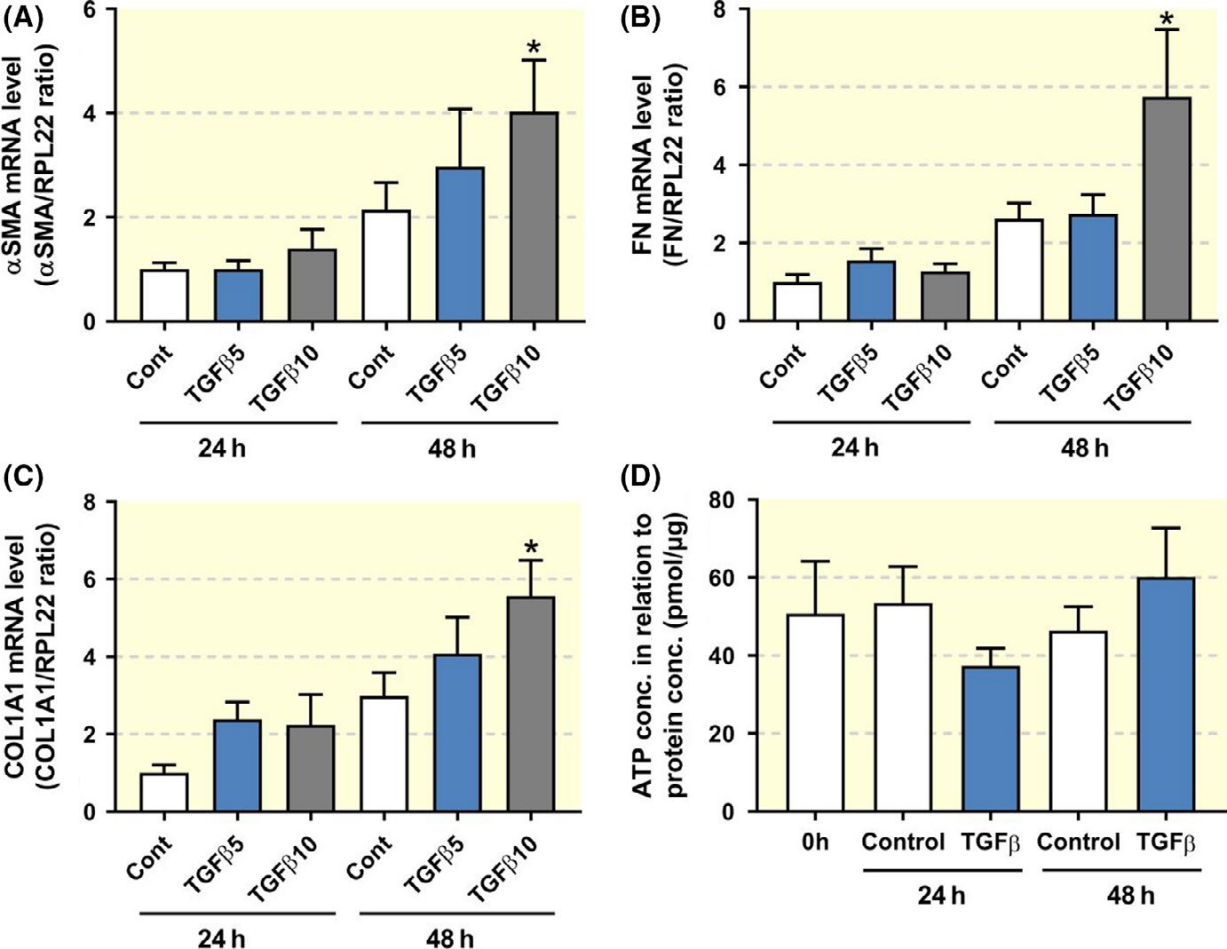
Expression of fibrosis markers in human PCKS. PCKS were exposed to TGF‐β (5 or 10 ng/ml) for 24‐48 h. (A‐C) Gene expression was studied by qPCR. Relative expression was calculated using the reference gene RPL22 (n = 4‐5). (D) Viability of PCKS after treatment with 10 ng/ml TGF‐β, assessed by ATP content of the slices (n = 5‐7). Data are presented as mean ± SEM. *P < 0.05

**Figure 7:**
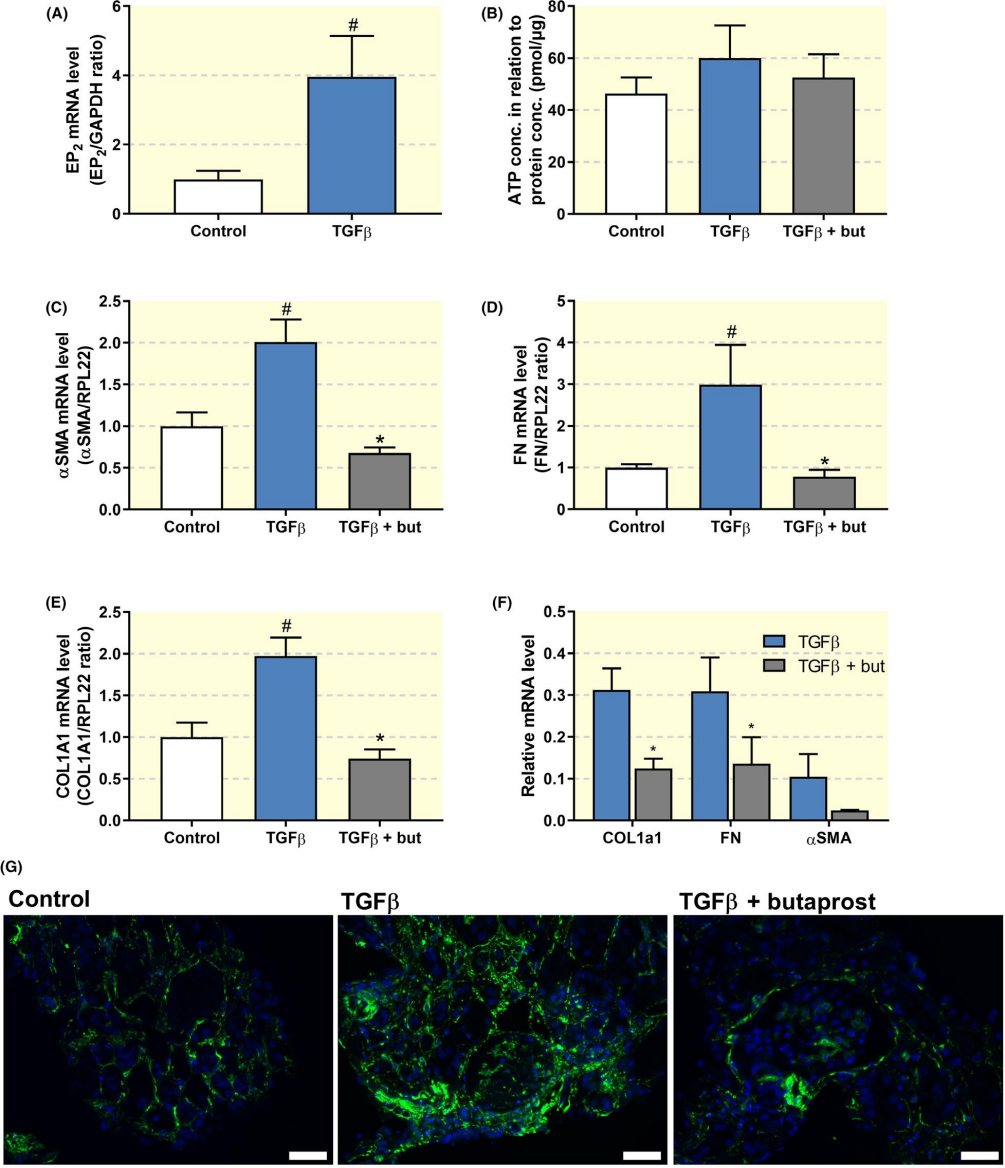
Butaprost attenuates TGF‐β‐induced fibrosis in human PCKS. PCKS were exposed to 10 ng/ml TGF‐β in the absence or presence of butaprost (50 μM) for 48 h. (A) EP_2 _receptor gene expression was studied by qPCR. Relative expression was calculated using the reference gene GAPDH (n = 4). (B) Viability of the slices after treatment assessed by ATP content of the slices (n = 7). (C‐E) Gene expression of fibrosis markers was studied by qPCR. Relative expression was calculated using the reference gene RPL22 (n = 7). (F) PCKS were also incubated with butaprost in the presence of indomethacin (10 µM), an inhibitor of both COX‐1 and COX‐2, to mitigate the influence of endogenous prostaglandins. (G) Representative images of immunolabeling for αSMA (green) counterstained with DAPI (blue). 20× magnification, scale bar is 50 μm. Data are presented as mean ± SEM. # and *P < 0.05 compared to control or TGF‐β, respectively

Finally, we investigated whether the anti‐fibrotic effect of butaprost could also be observed in a novel translational fibrosis model, *viz*. human PCKS. As shown in Figure 6, treatment with 10 ng/ml TGF‐β for 48 hours induced a fibrotic response in the slices, resulting in a more than fourfold increase in the gene expression of COL1A1, FN and αSMA (Figure 6A‐C), without affecting PCKS viability as evaluated by ATP measurements (Figure 6D). In addition, exposure to TGF‐β increased mRNA levels of the EP_2_ receptor, in line with the results obtained in UUO mice (Figure 7A). Moreover, qPCR revealed that butaprost significantly antagonized TGF‐β‐induced fibrogenesis, as illustrated by a reduced expression of all tested fibrosis markers (Figure 7C‐E) without affecting PCKS viability (Figure 7B). To exclude the possibility that endogenous prostaglandins elicited the anti‐fibrotic effects contributed to butaprost we performed several experiments in the presence of indomethacin, an inhibitor of both COX‐1 and COX‐2. Our results demonstrated that butaprost, in the absence of endogenous prostaglandins, still attenuated TGF‐β‐induced fibrogenesis (Figure 7F). In addition, fluorescence microscopy showed stronger αSMA staining in PCKS exposed to TGF‐β. Administration of butaprost and TGF‐β in combination diminished staining intensity as compared to treatment with TGF‐β alone (Figure 7G). Thus, butaprost also attenuates TGF‐β‐induced fibrogenesis in a human model of renal fibrosis.

## 3 DISCUSSION

Renal fibrosis plays a pivotal role in the development and progression of CKD as well as in renal transplant failure. As a result, many strategies have been developed in the hope of slowing down or even reversing the fibrotic process. Even though several studies have been successful at the pre‐clinical level, only limited advances have been made in the translation of these findings to the level of patient treatment.^11^ The search for effective therapies is mainly hampered by the absence of relevant translational models of renal fibrosis. Here, we investigated the anti‐fibrotic efficacy of butaprost, a selective EP_2 _receptor agonist, using various renal fibrosis models including a recently developed human model of the disease, *viz.* PCKS.

Using a bottom‐up translational approach, we demonstrated that butaprost successfully mitigated fibrogenesis in MDCK cells, UUO mice and human PCKS. To date, only a few studies have demonstrated renal protective effects of butaprost on a cellular level and, to the best of our knowledge, we are the first to unveil the positive effects of butaprost in a multicellular human PCKS model as well as in an in vivo model. On a cellular level, Liu and colleagues described that butaprost treatment prevented TGF‐β‐induced injury in MPC5 mouse podocytes, as illustrated by an increased proliferation and expression of slit diaphragm genes (nephrin, podocin and CD2AP), as well as a reduction in apoptosis.^12^ In addition, it has been demonstrated that butaprost reduced TGF‐β‐induced proliferation of glomerular mesangial cells, thereby diminishing renal injury.^13^ Evidently, butaprost elicits protective effects in various renal cell types.

In our hands, butaprost attenuated TGF‐β–induced EMT in MDCK cells. Even though the contribution of EMT to fibrosis remains a subject of debate, phenotypic alterations reminiscent of EMT, also referred to as epithelial phenotypic changes, do play a role in the development of renal fibrosis.^14^, ^15^, ^16^

The beneficial effect of butaprost is not limited to the kidney. Several studies have reported that butaprost also protects against pulmonary fibrosis. Kolodsick and colleagues demonstrated that butaprost attenuated TGF‐β–induced myofibroblast transition of IMR‐90 cells.^10^ In addition, butaprost has been shown to inhibit TGF‐β–induced CCN2/CTGF expression in lung fibroblasts.^17^ Furthermore, it has been demonstrated that butaprost reduces collagen synthesis in rat pulmonary fibroblasts and mitigates differentiation into myofibroblasts.^18^ Conjointly, these data indicate that butaprost appears to be a promising candidate drug for the treatment of organ fibrosis.

It is well known that stimulation of the EP_2_ receptor leads to activation of the cAMP/PKA pathway, and also in our hands butaprost increased cAMP levels in MDCK cells. However, our results indicated that the anti‐fibrotic effect of butaprost is independent of cAMP/PKA signalling. Interestingly, it has previously been demonstrated that cAMP is not necessary for butaprost‐mediated aquaporin‐2 membrane targeting, which was thought to be a cAMP‐dependent event,^19^ indicating that activation of the EP_2_ receptor might also affect other pathways. Our results further revealed that butaprost attenuated fibrosis by hampering TGF‐β/Smad2 signalling. This observation is in line with the study by Neil et al, showing that PGE_2_ reduced Smad3 expression and nuclear accumulation in normal and malignant mammary epithelial cells.^20^ Moreover, it has been demonstrated that EP_2 _mediates the suppressive effect of COX‐2 and PGE_2 _on TGF‐β‐induced Smad2/3 signalling in normal and malignant mammary epithelial cells as well as in Balb/C mice with mammary tumours.^21^ In addition, using human renal glomerular mesangial cells, it has been shown that PGE_2 _induced post‐translational modification of Smad2 and promoted Smad2/4 complex formation.^22^ These findings support the notion that EP_2 _receptor activation can directly influence TGF‐β/Smad signalling.

Here, we show that the EP_2 _receptor is expressed in interstitial myofibroblasts following UUO. The current understanding regarding the tissue distribution of the EP_2 _receptor is very limited. Using Northern blot analysis of mRNA expression, it has been demonstrated that the receptor is mainly present in the uterus, lung and spleen, exhibiting only low mRNA levels in the kidney.^23^ In rabbit kidney, the receptor was detected in glomeruli, thin descending limbs of Henle's loop as well as medullary and cortical collecting ducts.^24^ In the same study, it was reported that EP_2 _receptor mRNA could be detected in cultured renal medullary interstitial cells.^24^ In addition, in rat kidney, it has been shown that the EP_2_ receptor is mainly expressed in the descending thin limb of the loop of Henle and the vasa recta of the outer medulla.^25^ The described tissue expression is in line with the main function contributed to the EP_2_ receptor, namely renal salt and water handling. However, the increased expression observed during injury and the presence of the receptor in activated fibroblasts, as shown in this study, suggest an additional role in renal protection.

Stimulation of the EP_2_ receptor causes vasodilation and increases renal blood flow (RBF).^8^ It is known that preservation of RBF can diminish UUO‐induced renal fibrosis.^26^ Therefore, it is possible that improving RBF is one of the mechanisms underlying the renoprotective action of butaprost in vivo. Still, the main mechanisms of action seem to be a direct impact on TGF‐β/Smad signalling since the anti‐fibrotic effect of butaprost was also clearly observed in models lacking RBF, *viz.* MDCK cells and hPCKS.

Our findings provide the first preclinical evidence that targeting the EP_2_ receptor may prevent renal fibrosis, as such, the use of specific EP_2_ agonists may reduce the occurrence of cardiovascular and renal side effects associated with systemic targeting of COX‐2.

In conclusion, this study demonstrates that stimulation of the EP_2_ receptor effectively mitigates renal fibrogenesis in various models of kidney injury, mainly by targeting TGF‐β/Smad signalling. These findings warrant further research into the clinical application of butaprost, or other EP_2_ agonists, for the treatment of renal fibrosis.

## 4 MATERIALS AND METHODS

### 4.1 Ethics statement

The use of human tissue for the preparation of PCKS was approved by the Central Denmark Region Committees on Biomedical Research Ethics (Journal number 1‐10‐72‐211‐17) and The Danish Data Protection Agency. All participants gave written informed consent.

All animal experiments were performed according to the Danish National Guidelines for animal care, and were approved by the Danish veterinary and food administration (Approval no. 2015‐15‐0201‐00658).

### 4.2 Cell culture

MDCK epithelial cells were used to evaluate the anti‐fibrotic efficacy of butaprost (Cayman, Cat. 13741) in vitro. The cells were grown in Dulbecco's Modified Eagle Medium containing 10% foetal bovine serum and 1% penicillin/streptomycin. Cells were cultured at 37°C in a 5% CO_2_ atmosphere. Before experiments, cells were grown to 80% confluence and then serum starved for 24 h. During experiments, the cells were treated for 24 h with TGF‐β (5 ng/ml), butaprost (10, 20 or 50 µM), the adenylate cyclase inhibitor SQ22536 (75 µM), the protein kinase A inhibitor H89 (10 µM) or a combination hereof. Concentrations were based on previous dose‐finding studies performed in our lab. Butaprost and the inhibitors were added to the culture medium 30 min prior to exposure with TGF‐β.

### 4.3 cAMP levels

MDCK cells were cultured with and without TGF‐β and butaprost for 24 h, during the last 30 min the phosphodiesterase inhibitor IBMX (0.5 mM; Sigma) was added. Afterwards, cells were lysed and intracellular cAMP levels were measured using a cAMP enzyme immunoassay kit (Sigma) according to the manufacturer's instructions. All measurements were performed in triplicate.

### 4.4 Experimental animals

Experiments were performed using male C57BL/6 mice, 8 weeks of age and weighing 21 ± 2 g (Janvier Labs, Le Genest‐Saint‐Isle, France). All animals had ad libitum access to standard rodent chow (Altromin, Lage, Germany) and tap water. During the experiments, mice were housed in groups of 2‐3 mice/cage in a 12 h:12 h light‐dark cycle at a temperature of 21 ± 2°C and a humidity of 55 ± 5%. The animals were allowed to acclimatize to their cages 3‐4 days prior to surgery. A preliminary dose‐response study, using the following doses 1, 2 and 4 mg/kg/day, was performed using 4 animals per group. Subsequently, the anti‐fibrotic effect of butaprost was validated in a larger cohort, as described below.

### 4.5 Experimental design and surgical procedures

During surgery, mice were anesthetized with sevoflurane and placed on a heating pad to maintain an appropriate body temperature (37‐38°C). Through a midline abdominal incision, the left ureter was exposed and occluded with a 6‐0 silk ligature. UUO was maintained for 7 days. A total of 30 mice were divided into 4 experimental groups: sham‐operated (n = 6), sham‐operated receiving butaprost (4 mg/kg/day; n = 6), 7‐day UUO receiving intraperitoneal saline injections (n = 8) and 7‐day UUO treated with butaprost (4 mg/kg/day; n = 10). Butaprost, diluted in saline, was administered twice daily via intraperitoneal injection starting at the day of the surgery. Dosing was based on previous dose‐finding studies performed in our lab. After 7 days, the kidneys were extracted and blood was collected via cardiac puncture for further analysis. Biochemical analysis of blood samples was performed using a Roche Cobas 6000 analyzer (Roche Diagnostic) and creatinine levels were determined using the Creatinine Assay Kit (Sigma), according to the manufacturer's instructions.

### 4.6 Precision‐cut kidney slices

PCKS were prepared from functional (*ie,* eGFR > 60 ml/min/1.73 m^2^) and macroscopically healthy renal cortical tissue obtained from both male and female patients following tumour nephrectomies, as described previously.^27^ In short, slices were prepared in ice‐cold Krebs‐Henseleit buffer, supplemented with 25 mM D‐glucose, 25 mM NaHCO_3_, 10 mM 4‐(2‐hydroxyethyl)piperazine‐1‐ethanesulfonic acid and saturated with carbogen (95% O_2_, 5% CO_2_), using a Krumdieck tissue slicer. Subsequently, PCKS were cultured in William's E medium with GlutaMAX containing 10 mg/mL ciprofloxacin and 2.7 g/L D‐(+)‐Glucose solution at 37°C in an 80% O_2_, 5% CO_2_ atmosphere while gently shaken. Medium was refreshed every 24 h. PCKS viability was assessed by determining the ATP content of the slices using the ATP Colorimetric/Fluorometric Assay Kit (Sigma), according to the manufacturer's instructions. Patient demographics are presented in Table 2.

**Table 2:** Patient demographics

Gender (%male)	71.4
Age (years)	72.4 ± 5.3
BMI	24.5 ± 2.2
eGFR (ml/min/1.73 m^2^)	82.6 ± 7.5
Ischemia time (min)	42 ± 15

Values are presented as mean ± SD (n = 7).

BMI, body mass index; eGFR, estimated glomerular filtration rate.

### 4.7 Western blotting

Total protein was extracted using either M‐PER mammalian protein extraction reagent (cells) or RIPA buffer (kidney tissue), both supplemented with phosphatase‐inhibitor 2 and 3 and a mini protease inhibitor tablet. Afterwards, 2% SDS and DTT were added to the samples, and they were heated for 15 min at 65°C. Total protein was separated by SDS/PAGE using 12% Criterion TGX Stain‐free gels and subsequently blotted onto a nitrocellulose membrane. Afterwards, the membrane was blocked for 1 h with 5% skimmed milk in PBS‐T. The blot was then incubated overnight at 4°C with specific primary antibodies (Table 3). Afterwards, the membrane was washed with PBS‐T and incubated with the appropriate secondary antibody for 1 h at RT. Binding of the antibodies was visualized using ECL‐prime.

**Table 3:** Primary antibodies

Target	Catalog nr	Company	Species	Dilution
αSMA	M0851	Dako	Mouse	1:1000
FN	ab2413	Abcam	Rabbit	1:1000
EP_2_	ab167171	Abcam	Rabbit	1:500
Smad2	5339	Cell Signalling	Rabbit	1:1000
pSmad2	3108	Cell Signalling	Rabbit	1:1000

### 4.8 Immunolabeling

Kidneys were fixed by perfusion through the left ventricle using 4% paraformaldehyde (PFA) in water. Afterwards, kidneys were immersed in 4% PFA for 1 h, rinsed with PBS, dehydrated using a graded series of alcohol and embedded in paraffin. Subsequently, tissue sections (2 μM) were deparaffinized, rehydrated, and then boiled in TEG‐buffer for 16 min for epitope retrieval. Hereafter, sections were left to cool, and then incubated for 30 min in 50 mM NH_4_Cl to block free aldehyde groups. Afterwards, sections were incubated with blocking solution (PBS containing 1% BSA, 0.2% gelatin and 0.05% saponin) for 30 min. For immunoperoxidase labelling, the sections were incubated with a primary antibody against EP_2_ (Table 3) diluted in PBS with 0.1% BSA and 0.3% Triton‐X‐100 overnight at 4°C. Subsequently, the sections were washed three times with PBS containing 0.1% BSA, 0.2% gelatine and 0.05% saponin followed by incubation with a P448 secondary antibody diluted in washing solution for 1 h at RT. Afterwards, the sections were rinsed with PBS wash‐buffer, and the sites of antibody‐antigen reactions were visualized with 0.05% 3,3′‐diaminobenzidine tetrachloride (Kem‐En‐Tec, Copenhagen, Denmark) dissolved in distilled water containing 0.1% H_2_O_2_. Light microscopy was performed using an Olympus BX50 light microscope and CellSens imaging software.

For immunofluorescence labelling, sections were covered with mouse‐on‐mouse blocking solution containing unconjugated AffiniPure Fab Fragment Donkey Anti‐Mouse IgG (Jackson ImmunoResearch) in PBS for 1 h at RT and then fixed for 10 min in 4% PFA. Sections were incubated overnight at 4°C with primary antibodies (EP_2_ and αSMA, Table 3) diluted in PBS containing 0.1% BSA and 0.3% Triton X‐100. Sections were subsequently washed for 30 min in PBS containing 0.1% BSA, 0.2% gelatine, and 0.05% saponin and then incubated with Alexa Fluor 488 and Alexa Fluor 568‐conjugated secondary antibody at RT for 30 min (Life Technologies). Then, sections were counterstained with 4,6‐diamidino‐2‐phenylindole (DAPI), washed with PBS, and mounted with SlowFade Gold Antifade Mountant (Life Technologies). Fluorescence microscopy was performed using an Olympus BX61 microscope and image processing was performed using Xcellence Rt software.

In addition, sections were stained with hematoxylin and eosin to assess kidney damage and tubular dilation. In order to evaluate tubule volume, a grid overlay was placed on each picture and tubules located at the points of intersections were marked. Afterwards, the lumen was measured in percentages of the marked area. Five pictures were captured in a blinded manner from each specimen at x20 magnification with no overlapping regions, and 6 tubules were assessed in each picture. Interstitial volume was calculated using ImageJ software based on signal intensity of the αSMA immunolabeling.

### 4.9 PCR

Total RNA was isolated using either TRIzol Reagent (cells) or a NucleoSpin RNA II mini kit (kidney tissue; Macherey Nagel), following the manufacturer's instructions. RNA was quantitated by spectrophotometry and stored at −80°C. cDNA was synthesized from 0.5 μg RNA with the RevertAid First Strand synthesis kit (Thermo Scientific). To confirm expression of the EP_2_ receptor in MDCK cells, RT‐PCR was performed with (+) or without (−) reverse transcriptase (RT) enzyme. Afterwards, the PCR product was analysed by electrophoresis using a 1% agarose gel run at 70 V for 45 min, including a marker (Generuler DNA marker, Invitrogen). Images of the gel were obtained with an Azure c200 gel imaging workstation. To study the expression level of the other genes of interest, qPCR was performed using 100 ng cDNA, which served as the template for PCR amplification using the Brilliant SYBR Green qPCR Master Mix (Thermo Scientific), according to the manufacturer's instructions. Used primers are listed in Table 4.

**Table 4:** Primer sequences

Target gene	Accession number	Forward	Reverse
*Murine*			
αSMA	NM_007392.3	5′‐CTGACAGAGGCACCACTGAA‐3′	5′‐CATCTCCAGAGTCCAGCACA‐3′
FN	NM_010233.2	5′‐AATGGAAAAGGGGAATGGAC‐3′	5′‐CTCGGTTGTCCTTCTTGCTC‐3′
EP_2_	NM_008964.4	5′‐ATGCTCCTGCTGCTTATCGT‐3′	5′‐AGGGCCTCTTAGGCTACTGC‐3′
COL1A1	NM_007742.4	5′‐CACCCTCAAGAGCCTGAGTC‐3′	5′‐ACTCTCCGCTCTTCCAGTCA‐3′
18S	NM_011296.2	5′‐GAAAATAGCCTTCGCCATCA‐3′	5′‐TCCCATCCTTCACATCCTTC‐3′
*Human*			
EP_2_	NM_000956.3	5′‐CCACCTCATTCTCCTGGCTA‐3′	5′‐TTCCTTTCGGGAAGAGGTTT‐3′
αSMA	NM_001141945.2	5′‐ACCCACAATGTCCCCATCTA‐3′	5′‐GAAGGAATAGCCACGCTCAG‐3′
FN	NM_212482.2	5′‐CAGTGGGAGACCTCGAGAAG‐3′	5′‐GTCCCTCGGAACATCAGAAA‐3′
COL1A1	NM_000088.3	5′‐CCTGGATGCCATCAAAGTCT‐3′	5′‐AATCCATCGGTCATGCTCTC‐3′
RPL22	NM_000983.3	5′‐TCGCTCACCTCCCTTTCTAA‐3′	5′‐TCACGGTGATCTTGCTCTTG‐3′
GAPDH	NM_002046.5	5′‐ACCAGGGCTGCTTTTAACTCT‐3′	5′‐GGTGCCATGGAATTTGCC‐3′
*Canine*			
TGFβ	NM_001003309.1	5′‐AAGAAAAGTCCGCACAGCAT‐3′	5′‐GCTGCTCCGCTTTTAACTTG‐3′
GAPDH	NM_001003142.2	5′‐AACATCATCCCTGCTTCCAC‐3′	5′‐GGCAGGTCAGATCCACAACT‐3′

### 4.10 Statistics

Statistics were performed using Graphpad Prism by either one‐way ANOVA followed by Tukey's or Dunnett's multiple comparisons test, two‐way ANOVA followed by Tukey's post hoc test or using an unpaired two‐tailed Student's t test as appropriate. Differences between groups were considered to be statistically significant when *P* < 0.05.

## CONFLICT OF INTEREST

The authors have declared that no conflict of interest exists.

## AUTHORS' CONTRIBUTIONS

MSJ, HAMM and RN designed the study; MSJ, HAMM, SJT, MC, TK and RN carried out experiments and analysed the data; PO provided additional analytical tools and chemicals for this study; MGM helped with human tissue procurement; MSJ, HAMM and RN wrote the manuscript with critical review from SJT, MC, MGM, PO and TK. All of the authors approved the final version of the manuscript for publication.
